# Ozempic and Wegovy Meet Congress: Political Economy Without Critique and Sociology Without Society

**DOI:** 10.1177/27551938261425193

**Published:** 2026-02-26

**Authors:** Rodney Loeppky

**Affiliations:** 1 Department of Politics, 7991 York University, Toronto, Canada

**Keywords:** political economy, medicalization, health care, pharmaceutical industry, obesity

## Abstract

In the fall of 2024, a U.S. Senate Hearing on the high price of Novo Nordisk's prescription drugs Ozempic and Wegovy underlined some of the deepest challenges in the U.S. health care system. This article utilizes critical approaches to both political economy and sociological discussions of medicalization as a means to address these challenges. Market-led organization and medicalization underlie not only these products’ pricing, but also wider issues of cost control and health care organization. Congressional debate tends to either attribute blame on individual corporate actors, largely sidestepping larger structural issues, or defend industry and profit-oriented care, deflecting blame for high costs on the lifestyle choices of individual Americans. The article reestablishes an interrelation between critical political economy and medicalization, calling for a reorientation of health purchasing in the United States, along the lines of a single payer system that would, by necessity, interrogate the value of evolving drug (and other medical) provision for American society. This argument has strong practical political implications, potentially enabling policymakers to approach the emergence of ‘wonder’ drugs like Ozempic/Wegovy on terms that matter to both patients and U.S. society—price, medical necessity, access, and overall health care costs.

On September 24, 2024, the U.S. Senate Committee on Health, Education, Labor and Pensions (HELP) held hearings on the high price of diabetes and weight-loss drugs, Ozempic and Wegovy. These blockbuster drugs, with identical active ingredients, have been heralded as breakthrough treatments for type II diabetes and obesity, respectively.^1–3^ With single testimony given by Lars Jorgensen, then-chief executive officer (CEO) of the drug's manufacturer, a Democrat-led committee sought to lower prices and expand access for American patients. While much has changed in a second Trump administration, particularly with the dramatic health care cutbacks of Republicans’ reconciliation legislation,^4^ both parties continue to declare the need to lower pharmaceutical pricing. But the pathway to such an outcome remains highly contested, where cost control is in no way a settled process.

In this way, the HELP hearing on Ozempic/Wegovy opened a window on some of America's deepest health care divisions, mapping out contending political positions and strategies. As such, this study asks why, in the face of ostensible bipartisan political support, meaningful progress on excessive pharmaceutical price levels remains so difficult? It argues that two broad dynamics in the U.S. system—market-led organization and medicalization—underlie not only the controversy related to Novo Nordisk's products, but also the most serious—and least addressed—shortcomings of American health care. In this regard, a critical political economy forms the main conceptual underpinning of the discussion, with the recognition of capitalism as *systemically* driven by the profit imperative, where producers are *compelled* to accumulate. In turn, such a political economy fits consistently with an accompanying framework of medicalization, typically located within sociological debate.^5–7^ Here, the primary concern has been the ever-growing boundaries of medicine, which parallel a greatly enlarged provision of highly profitable prescription drugs, where the necessity of their expanded use is not always obvious.^8^ Taking these concepts together, the article ultimately concludes that controlling spiraling pharmaceutical (and other) costs will require governmental intervention that utilizes centralized purchasing to counterbalance the manufactured complexity of U.S. health provision, and that such a process implies the critical reevaluation of societal health needs separated from the profit imperative.

The significance of this approach lies, first and foremost, in its interdisciplinary nature. Political economy and medicalization critiques are too often held apart, with only limited exceptions.^8,9^ This contribution reinforces a call for medicalization to go beyond moral or philosophical analyses of medical intervention, recognizing that structural conditions may well overcome such considerations.^10^ In a related sense, critical political economy moves away from a position in which the actions of particular agents—in this case Novo Nordisk—are critiqued as uniquely problematic or out of the ordinary. Rather, it focuses on what would be expected from that actor within prevailing structural conditions. This means that the Congressional tendency to seek out culprits for runaway costs within the health system sidesteps more trenchant structural questions regarding system organization and the normalized function of profit motive within health provision.^11–13^ It also puts in question Republicans’ utilization of medicalization to individualize health issues and responsibility regarding pharmaceutical usage while applauding the industry's profit motive. Without a more serious and interwoven understanding of these two dynamics, the ability to address issues raised by the emergence of ‘wonder’ drugs like Ozempic/Wegovy, price, medical necessity, access, and overall health care costs) will remain elusive.

## A Brief Note on Method

Grounded in critical political economy, this article seeks an explanation for corporate pharmaceutical behaviour based on the structural relations of capitalism. Ellen Wood argues that capitalism “is a system in which goods and services, down to the most basic necessities of life, are produced for profitable exchange . . . where all economic actors are dependent on the market . . . [and] the production of goods and services is subordinate to the production of capital and capitalist profit”.^14^^,pp. 2-3^ It is not monolithic in form—as with all social structures, it is sensitive to time and place. In this sense, after capitalism emerged in England, “it could never emerge again in the same way. Every extension of its laws of motion changed the condition of development thereafter, and every local context shaped the processes of change” (^14^, p. 176). In the American context, this has meant the full support of the U.S. government in a political culture that is typically more accepting of the imperatives of capital and the imposition of short-term profit outlook (^15^, p. 106). Oddly, this same political culture that heavily validates the open market and denounces government intervention is also deeply dependent on public revenues that shore up multiple industrial sectors.^16^ As we will see here, the pharmaceutical sector, in particular, has navigated this complicated terrain with extreme success.^17,18^

The use of critical political economy in relation to health cannot avoid the question of medicalization, a concept first utilized by sociologists to capture the expansion of the medical domain. An early critic, Irving Zola, characterized this as “‘medicalizing’ much of daily living, by making medicine and the labels ‘healthy’ and ‘ill’ relevant to an ever increasing part of human existence” (^19^, p. 210). While the health field has certainly become more complex, the central challenge outlined by Zola holds.^19^ And while a range of new concepts, including ‘pharmaceuticalization’ and ‘biomedicalization’, have entered the debate, their analytical purchase is not altogether clear.^20,21^ Peter Conrad is correct to assert the necessity to prioritize themes, and that the central problem in medicalization remains a definitional one.^10^ What conditions, what states of being, should properly be understood as constituting medical need and, therefore, require medical intervention? In this sense, “the definitional center of medicalization remains constant, but . . . commercial and corporate stake-holders play a major role in how the technology will or won't be framed”.^10^^,pp. 10-11^ Conrad thus calls on sociologists to focus their efforts on the prime mover in medicalization, calling for less fascination with technoscientific advance and more attention to the rather blatant material commercial interests that drive it.

In this regard, the role of the pharmaceutical industry in advancing medicalization has been highlighted for some time. Certainly the industry has always been the primary driver behind the now four-decade long explosion of biotechnological research and application.^22,23^ But it has also been involved in medicalization in ways that Zola would likely not have foreseen in the early days of the debate. Not long before Conrad was nudging sociologists to pay more attention to the political economy of medicalization,^10^ Ray Moynihan, Iona Heath, and David Henry suggested that pharma was ‘selling sickness’, largely through pushing the boundaries of illness in some areas and broadening them in others.^24^ They run through a range of human conditions, including baldness, irritable bowel syndrome, ‘social phobia’, osteoporosis, and erectile dysfunction, to demonstrate how drug companies tend toward disease-mongering. The authors highlight “. . . the invisible and unregulated attempts to change public perceptions about health and illness to widen markets for new drugs” (^24^, p. 890). This is not to suggest that pharmaceuticals do not play an important role in therapeutic intervention. Instead, the danger is a trend in which market-making firms control drugs and biologicals in a process that seeks a wider and more voluminous set of applicable conditions.^25–27^ While it is impossible to demand any objectively valid and exact dividing lines between ‘normal’ human conditions and ill-health, there are both iatrogenic and societal dangers in industry unnecessarily expanding medical need, or creating it where none might previously exist. The basis for such an expansion cannot merely be understood to be changing societal values, but instead is related to the material power relations associated to the health industry—a reality only accessed effectively through the combined lenses of political economy and medicalization.

## The Ozempic/Wegovy Senatorial Hearing

U.S. Congressional hearings are complicated affairs, with many purposes, but they are highly indicative and revealing of political positioning and intent.^28^ Called by the party in majority, authorizing the Chair to invite witnesses, they serve purposes of either governmental oversight or gathering information for committee members on important topics which may affect the legislative process. Beyond these formal purposes, however, hearings also regularly amount to questioning of witnesses by House Representatives or Senators on terms they find politically advantageous or appealing. Indeed, interventions by committee members can often be used to either embarrass or prop up witnesses, or they can be positioned as a foil for speechmaking. While this might be characterized as ‘political theater’, it nonetheless renders a picture of not only how parties understand salient issues, but how they position themselves, and what the outer boundaries of political action might be.^28^^,pp. 584-585^

The Ozempic/Wegovy hearing focused, first and foremost, on the issue of price. Then-Chairperson Senator Bernie Sanders (who is nominally an Independent but caucuses with the Democrats) opened the gathering by pointing to huge price differentials between Organisation and Economic Co-operation Development (OECD) countries and the U.S. on these drugs. They are, he argued, “on track to be some of the best-selling and most profitable drugs in the history of the pharmaceutical industry” (^29^, p. 3). Sanders estimated that if half of eligible U.S. patients took the drug Wegovy, it would have “a devastating financial impact on our country and on federal and state budgets. Best estimate . . . it would cost $411 billion a year, and that is more than what Americans spend on all prescription drugs . . . .” (^29^, p. 5) Having laid out the cost impact, the tenor of his questioning turned to the issue of individual corporate behaviour: “From a moral perspective, does it bother you knowing that keeping the price of Ozempic and Wegovy so high in the United States could lead to the preventable deaths of tens of thousands of Americans?” (^29^, p. 34) In fact, this appeal to a moral imperative underwrites much of the critique and questioning from Democrats. If the company has benefited enormously from the American market to date, why should it not now make these drugs (and others) more affordable, even if still profitable? The expectation that political embarrassment or shame will incentivize action on the part of the company assumes that economic agents will disregard structural conditions of the market or their sector, as long as there exists a persuasive enough political or moral argument.

Throughout much of the hearing, for instance, pharmacy benefit managers, PBMs) were highlighted as problematic intermediaries within the pharmaceutical production–distribution chain. PBMs negotiate rebates on behalf of insurers, garnering a rebate from the manufacturer, the latter of which grows with higher list prices.^30,31^ While initially conceived as a cost-saving mechanism between purchaser and provider, their position has become the subject of criticism, as PBMs have grown in size and leverage, have become vertically integrated with insurance actors, and do not disclose information on either the size of rebates or their ultimate distribution among actors (^32^, p. 405). The fear, according to Jorgensen, is that PBMs will drop drugs from their drug formularies if the list price is not lucrative enough, diminishing accessibility for U.S. patients. Focused on this, Democrats tried to extract a commitment from Jorgensen to lower prices, given previous pledges by individual PBMs not to block accessibility. Senator Maggie Hassan, for instance, sought to persuade (or gently cajole) Novo Nordisk into action: “So, I would strongly recommend that with these companies on the record . . . that you consider strongly lowering the list price” (^29^, p. 47). Here, with the moral argument in the background, political pressure is applied to shift individual corporate behaviour, with the assurance that their de facto revenue stream will not be affected.

For their part, Republicans engaged in the same critique of individual agents, redirecting blame onto PBMs, positioning them as the real source of high prices. This tracks with a historical inclination of Republicans to both validate the pharmaceutical industry and defend it against criticism.^15–17^ This means that the search for individual culpability is merely relocated, with Senator Roger Marshall asserting that, “Novo Nordisk is not the villain in this story! They’re a hero . . . Now we all agree on the committee and across the Senate that the cost of healthcare is too much, and that prescription drugs are too high, especially the out-of-pocket expenses. But we need to figure out who the villain is, who is the real culprit here. Who's making the money?”.^29^^, pp. 42-43^ This is followed by other moderate Republicans emphasizing the role of PBMs as middle-negotiators and minimizing the actual returns realized by pharmaceutical corporations, with one Senator using a pie graph to suggest that drug companies take in only 26% of their own revenue (^29^, p. 43). The hesitancy or unwillingness fully to confront the structural dynamics of the pharmaceutical sector in the context of an uncoordinated, market-led health care system of purchasing and provision is notable and striking.

Republicans further shifted attention away from systemic dynamics, by apportioning responsibility onto individuals in ways that sidestep industry practices. One problem, they argued, is that Americans want ‘drive-through health care,’ and, as a result, do not want to think about their own role in personal health. Here, representatives emphasized the importance of lifestyle choices leading to obesity, diabetes, or other overall health challenges. Senator Bill Cassidy suggested that “Americans need to look in a mirror, that nutrition is a big problem in this country and lack of activity . . . . It's frustrating . . . that Congress can spend a trillion dollars on the military, Medicare can spend a trillion dollars, but we can’t spend 3 billion dollars on primary care” (^29^, p. 44). Highlighting these issues positioned the pharmaceutical industry (along with other providers) as mere *reactive* agents in the provision process, but it also mobilizes a medicalization discussion to fend off criticism of industry practices of marketing and pricing. And even though Cassidy mentions the dilemma of pharmaceutical marketing in the same breath, Novo Nordisk's own role in enlarging the use of this drug remains completely unscrutinized—to say little of the wider role of corporate America in fostering the life ‘choices’ of Americans. Notably, the company has come under scrutiny for its marketing practices, including over-accentuating obesity as a disease, installing fear or false promise in relation to obesity and health outcomes, and inundating advertising channels, particularly across social media.^33,34^

Focusing on the moral compass of corporate agents, as well as redirecting responsibility for the societal costs of obesity onto individuals, allows for maneuverability on the part of pharmaceutical CEOs like Jorgensen. With the topic area parsed out in this matter, cost relief can be raised as a possibility on a case-by-case basis, addressing the morality question, while potential price *regulation* (as occurs in virtually every other OECD country) is depicted as dangerous, choking the industry and denying Americans early and extensive access to life-saving medicines. Similarly, medicalization-related discussions about lifestyle and medical needs create an opening to position corporate behavior as inherently tailored to the individual needs of patients. This avoids any integrated view of what would happen for American citizens/patients were a more systematized and universal form of price regulation (for both drugs and other components of medical provision) and health care access to take hold. Even the harshest criticisms emerging from the HELP hearing maintain—perhaps understandably—a problem-solving approach, usually encouraging industry-negotiated solutions to prevailing challenges within the existing system. The result is that industrial players retain decision-making power and Congressional members can claim an ostensible impact on the health and well-being of Americans while not upsetting the complicated array of connections between these political actors and a powerful and influential industry.

### Political Economy and the Compulsion of Market Production

The understandable desire of political figures to achieve cost control on drugs is plagued by a single challenge: U.S. political economy is a *system*, and its outcomes are not fundamentally altered by the actions of individual agents. U.S. health care is unendingly complex, with an extensive set of actors on both the purchasing and provision sides of care.^35^ Even on a regional basis, there is no single purchaser of provision who wields enough systemic leverage to rein in prices in any serious way. One strong virtue of a single-payer health insurance system is monopsony, where there exists only one buyer for health care provision. Providers tend overwhelmingly to take the price offered, and this holds costs at a lower level, though cost pressures never cease, as providers are, in the main, still market actors.^36^ Barring monopsonistic structure in purchasing, the only possibility for meaningful cost control remains the regulatory power of the state. This is the case, for example, in Germany, where a dense network of purchasers and providers works under the umbrella of socially embedded state regulation.^23,37^ The American state, however, has proven to be deeply resistant to both possibilities. The federal government has prohibited itself statutorily from cost control through both the Medicare Modernization Act (MMA) and the Affordable Care Act (ACA). In fact, for the first time, under the Biden Administration, the Inflation Reduction Act allowed federal Medicare to negotiate prices on a limited number of drugs, although any negotiations on an expanded number of significant drugs have now been put in question by the Trump administration.^38^

A central problem is that capitalism operates as a series of socio-structural imperatives for all of its functional actors.^14^ And while those in search of reform often turn to moral opprobrium as a pressure point to influence the behaviour of egregious actors, this does little to change the conditions in which corporations operate. Here, a critical political economy makes clear that the latter operate under the compulsion of the market, however mediated and regulated that market may be.^14^ The ensuing outcomes may be damaging to society, but these ‘capitals’ are *compelled* to maximize their competitive returns on threat of extinction—if they do not, they can be sure that their counterparts will.^39^ In limited ways, Republicans have understood this, hailing such drugs as Ozempic/Wegovy as successes of the marketplace, and suggesting that all the corresponding returns of the marketplace must properly follow.^40^ What they misunderstand are the many non-market inputs, which both generate the conditions for such ‘breakthroughs’ and enforce the extraordinary returns for products brought to market.^41^ These inputs run the gamut from publicly funded basic research to far-too-extensive patent protection mechanisms. Democrats, too, adopt this logic when they highlight the industry's peculiar ‘risk’ in the market but also seek pricing relief on particular medicines. This call for exemptions validates the sector's self-portrayal as champions of patient well-being in the face of extraordinary market and regulatory pressures, ceding important political ground that industry can and will utilize.

During the HELP hearing, after Senator Mike Braun suggested that a lack of transparency in pricing has left the industry ‘screwed up’, Sanders replied directly that it is not screwed up but, rather, doing exactly what it is supposed to do.^29^^,pp. 58-59^ Intended or not, Sanders was pointing to the overall systemic *imperative* that urges profits upwards and leaves patients/citizens with diminished (or no) access and burdensome costs.^42–44^ Over two decades ago, Arnold Relman and Marcia Angell pointed out the questionable claims of the drug industry in relation to their costs and profit levels.^45^ They took aim at the industry's highly inflated figures associated to research and development, its gaming of the patent system, deeply troubling marketing practices, and constant invocation of market ‘risk’ as the justification for extraordinary returns on investment. They argued that a “business consistently this profitable cannot by any stretch of language be described as ‘risky’ or as needing special protection of its revenues” (^45^, p. 30). Over the years, the grounds for critique may have shifted, focusing less on the industry's exorbitant marketing and more on stock buybacks, but the argument holds. Incessant claims of perilous sectoral risk do not square with consistently huge profits and profligate non-research spending.^18^ Stock buybacks and dividend compensation have far outpaced spending on research and development and, systemically, generate the most pressure on managerial performance in a shareholder-heavy capitalism.^46^ Sanders’ instincts regarding systemic intention remain on point: why would drug industry actors, including Novo Nordisk, not use every political and economic tool at their disposal within a production and distribution system that urges them to maximize returns?

In this regard, the lobby and political influence associated to pharmaceuticals appears to many as a firewall that protects and shores up a very advantageous industrial position.^47–49^ The numbers associated to this political ‘buy-in’ leave little room for doubt around the sector's political leverage. The industry hit peak lobbying and contribution levels in 2023, spending $383 million for access and influence in federal politics, including Novo Nordisk, which alone spent $5.2 million on lobbying.^50^ Novo Nordisk is a firm whose majority shareholder is a nonprofit foundation, dedicated to medical research.^51^ Nonetheless, as one Danish journalist insisted in relation to the company's runaway profit levels, “Of course it's—it's ‘greedy’. You have to compete in the world as it is, and I don’t think that Novo Nordisk has all these values just to be ‘nice’. They have it because it's good business”.^52^ And it has, indeed, been good business: from 2017, when Ozempic was first approved for sales, the company's gross revenues have climbed steadily from $14 billion to $28 billion in 2023.^53^ The skyrocketing utilization of the drug as a weight-loss intervention has made the prospects for sales tantalizingly large, reflected in the latest year-on-year gross profit report, a 28% increase on 2023, at $33 billion.^54^

This may draw strong political reactions from both parties over profit levels that are derived largely through U.S. sales, but the country has proven historically amenable as a terrain conducive to precisely such outcomes. In response to the increasing drug prices at the time, Congress passed the 1984 Drug Price Competition and Patent Term Restoration Act (Hatch-Waxman Act). Its nominal goal was to allow greater access of generics into the prescription drug market while also maintaining deference to the powerful pharmaceutical industry. It accomplished the former by not requiring repetition of the Food and Drug Adminstration (FDA) regulatory approval process for equivalent generic drugs. Simultaneously, however, it granted up to five years of patent restoration to research-derived products for time lost during approvals (^55^, p. 49). In both a statutory and political sense, this set the trajectory for the industry's utilization—and abuse—of monopolistic patent power over the course of the next four decades, as well as its endless love–hate relationship with the FDA and Patent and Trademark Office (PTO). Both of these organizations have garnered endless criticism from industry for their ostensibly slow-moving bureaucracies, while simultaneously providing them with the surety of predictable (and socially legitimate) safety standards and legal property protection. Subsequently, the MMA in 2003, a center piece of the Bush administration, not only created a market of lucrative pharmaceutical benefits for Medicare recipients (Medicare Part D), but also explicitly prohibited the purchase of cheaper drugs from Canada *and* disallowed any use of federal bargaining leverage to reduce drug prices.^56^ On the former, while drug imports from Canada were quantitatively not significant to the industry, the perception problem—advertised in the media—of Medicare recipients crossing the Canadian border in buses to fill their prescription needs, while individual states publicly considered cheaper, cross-border drug provision sources for their Medicaid programs, constituted enough of a threat to prompt industry reaction. Far more significantly, on the latter, the government tied its own hands in relation to the one regulatory power it had to rein in rising drug prices. Finally, the Obama administration followed suit with the ACA in 2010. This act not only incorporated the prohibition on price negotiation for the drug industry and hospitals, it also expanded the existing system of divided, market-led health care purchasing and provision. This greatly enlarged available markets for insurance companies, drug companies, and PBMs, and it further entrenched a complicated pattern of provision and distribution that is now very difficult to untangle.^57,58^

These three legislative events, by no means an exhaustive picture, do give an indication of the interlocking structures (trade law, regulatory functions, health care policy) that firms like Novo Nordisk have at their disposal. While some of these, such as patent law, are similar in other countries, their ultimate effects are curtailed elsewhere by profound differences in surrounding regulatory and health care policy. In Canada, for instance, the combination of regulatory price control and bulk purchasing by provincial health plans has meant that per capita spending in 2021 was $814 versus $1432 in the United States (^59^, p. 1349). Even without a full-fledged national pharmacare program, Canadian drug prices are 44% of U.S. prices (^60^, p. VII). Indeed, taking a wider comparative lens, “U.S. manufacturer gross prices for drugs in 2022 were 278% of prices in the 33 OECD comparison countries combined. Put another way, prices in other countries were 36%—or a little more than one-third—of those in the United States” (^60^, p. 5). Pharmaceutical firms have navigated the U.S. regulatory and purchasing terrain deftly, maximizing their returns, given the specificity of constraints and opportunities that are statutorily available to them. Elsewhere, this dynamic has been labelled ‘adaptive accumulation’, where capitalist actors harness existing state structures to maximize their returns in ways that are specific to political geography.^16^ In this way, the expectation within Congress that firms will cede ground to competitors for reasons of morality or societal well-being hints at a certain politico-economic naïveté. Novo Nordisk, like other companies, will not fail to defend its profit margins or its return to shareholders unless structurally forced to do so.^61,62^ This would mean, among other things, changing the structure of U.S. health care coverage and cracking open the shell game of list price versus net price.

## Republican Revival of the Medicalization Debate

In a twist of ideological and political positioning, the long-standing debate on medicalization, highlighted here, has been imported by Republicans to address discussions of drug costs. As the hearing makes clear, political figures on the right suddenly find themselves attentive to the food consumption and physical activity patterns of their constituents. In the case of Ozempic/Wegovy, the implication of such interventions is that blame should not be apportioned to a corporate entity that is merely trying to meet existent needs in the marketplace. Rather, if these drugs signal an exorbitant cost for the U.S. health care system, it is because too many Americans seek quick medical solutions to enduring personal problems. On one level, this argument is a political maneuver to divert attention away from sky-high prices and unique U.S. profit levels. Simultaneously, however, it also harnesses a medicalization critique, typically undertaken by the critical left,^9,63,64^ but more recently utilized by anti-scientific cynics across the Republican party and much of its voting public.^65^ This is worth untangling, not the least because medicalization, when taken more seriously, relates both to the cost of U.S. care and its systemic organization.

On the issue of obesity, scholars of medicalization (among others) have strongly taken issue with both its cultural circulation and political targeting.^66–69^ Within this debate, Novo Nordisk currently attributes to itself an almost heroic, savior-like status. It has partially adopted the language of obesity activists, to claim that anybody questioning the widespread distribution of Wegovy is simply shaming victims for a medical problem. Jorgensen's opening statement makes this abundantly clear, where he opines that individuals “suffer from a false-yet-persistent social stigma that obesity is a moral failing or a lack of willpower . . . . This narrative has permeated public discourse for decades and has unfairly stigmatized patients for what we now know is a complex, chronic, and treatable disease (^29^, p. 14). Aligning the disease model with an anti-stigma position regarding obesity is hardly without controversy. It distills complex critiques of societal norms into simplified individual empowerment or suffering, based on degree of access to medical intervention. Indeed, “a discourse has now emerged in which opposition to the pharmaceuticalization of ‘obesity’ is labelled stigmatizing by clinicians or ‘anti’-obesity affiliates, even when pharmaceutical conflicts of interest are present” (^70^, p. 858). Stigmatization certainly does not disappear with the appearance of a medical diagnosis, as patients of cancer, HIV/AIDS, or addiction well know.^71–73^ More importantly, however, there is no consensus on the medicalization of adiposity, leading researchers to question whether it is doing more harm than good.^67^ By discounting the societal and environmental factors associated to weight, as well as jettisoning any meaningful interrogation of social norms and prejudices, the alliance between pharmaceutical intervention and weight loss may recreate the very problem it is ostensibly seeking to solve. It does nothing to challenge the behavioural approach to weight loss, merely shifting blame to those who fail to avail themselves of the latest ‘treatments.’ This, in turn, is “unlikely to disrupt weight-based stigma. However, it may sell more pharmaceuticals, while deemphasizing approaches that encourage self-acceptance and health optimization through self-care or ones that champion rectifying social inequities” (^66^, p. 746).

It is in this sociological context that we should interrogate the confusing nature of Republicans’ intervention. As with so many other areas of political controversy, Republicans have turned the critical debate involving medicalization on its head.^74^ In their hands, it amounts to a celebration of the market-based distribution of high-priced, patent-protected pharmaceuticals, *combined with* a punitive turn towards responsibility and blame, suggesting a failure on the part of individual Americans to recognize their own reliance on ‘drive-through health care’.^29^ In the mid-1990s, an intervention by Simon Williams and Michael Calnan—two British sociologists, long active in the medicalization debate—suggested that an increasingly cynical ‘lay populace’ would be able to critically assess and navigate their own medical needs, and that individual rationality and decision making could counter (or limit) the impact of medicalization.^75^ The logic of their argument could be understood as consistent with current Republican thinking on the role of medicine in society (^75^, p. 1616). The unspoken dilemma for Republicans is that the same biomedical forces towards which they have encouraged rational doubt are also central to a highly profitable health-industrial sector which they fully support.^76,77^ As such, they use medicalization critiques to reconcile this contradiction, by displacing culpability from the health industry onto individuals’ behaviour and problematic usage of health care.^78^ Calnan and Williams’ ‘lay populace’, in other words, need their individual decisions rerouted, such that costs for pharmaceutical care can be reined in, but market-led health can proceed unimpeded.

## Reconsidering Medicalization and Political Economy: Who's Afraid of Rationing?

Republican strategies around health care carry a degree of political risk. When the party tried to repeal the ACA in the first Trump administration, it paid a heavy political price in the midterm elections. The reasons the ACA has become so politically resilient are related to its institutional embeddedness, both with the wider American public and corporate participants.^79^ Democrats have increasingly understood this, as they sought, during and after the pandemic, to expand its reach across the populace as much as seemed politically feasible.^80^ At the time of the hearing, Republicans did not want to appear to be taking away access to health care in general, or to a particular treatment like Ozempic/Wegovy, but they do not fail to weigh in on the side of industry, either. Their default response is often to dismiss anything ostensibly not attuned to market signals. Senator Mitt Romney exemplified this by referring to the ‘fever-dream’ of regulated prices under capitalism: “Sometimes we live in a fantasy land, which is we want . . . the industry generally to invest massive amounts of money, but then we want you to keep the prices low . . . like, that's fantasy land, that's not reality . . . . There are alternative systems, which is . . . no, no, we’re gonna limit what you can get back. I look around the world—I don’t recall a lot of drugs coming from China and Russia and North Korea and Iran”.^29^ Here, the menace of communism is equated with any form of regulation or state involvement that might interrupt the industry's extraordinary profit levels. The fact that robust capitalist economies exist in every other OECD country alongside state-regulated health care and drug pricing is of no consequence to Republicans.^23^

Indeed, a kind of ‘red scare’ argument, still ideologically powerful, is regularly mobilized against even the hint of universalizing trends in health care. Government involvement that interferes with maximal profit-taking almost immediately invokes images—spoken or not—of central command economies, with empty store shelves and lineups for staple consumer items.^81,82^ Should the possibility of price regulation of the drug industry begin to emerge, alongside the previously mentioned, problematic arguments about the ‘risk’ associated to pharmaceutical research and development, the specter of ‘rationing’ is almost immediately raised, with corporations obstructed from performing their ostensible primary and desired goal: meeting the *needs* of patients/consumers. Jorgensen certainly made this clear in the HELP hearing when he pointed out that other OECD countries that do regulate pricing suffer from delayed and, ultimately, limited access to their drug. Merely mentioning the term *rationing* in the American political context taps into a deep suspicion of government as a societal actor and runs counter to prevailing market fundamentalism.^83^

Importantly, though, rationing can be productively related to medicalization discussions. Once dislodged from uncritical arguments that dogmatically praise the market as the source of all innovation, production, and distribution, medicalization discussions can constitute the substantive basis for decisions around the boundaries (rationing) of a society's medical needs.^84,85^ These discussions would have to be broad-ranging to capture not only scientific factors but also the value of evolving societal norms and expectations. They would ask what kinds of medical intervention correspond to medical need, how elastic the understanding of medical need should be, and how to reasonably estimate the value of any intervention. These questions may seem to be overly-searching for any regulator, but most societies do this as a matter of course—even in market-led health care systems.^86–88^ In fact, rationing is already a negative reality in American health, whether recognized by patients and practitioners or not. It is largely disguised, because it exists either in the counterfactual world of ‘no coverage’ or in myriad decisions take by health insurers.^89^ In Medicare Advantage, a privately run, government-funded program which now comprises over half of the Medicare population, insurance corporations seek out the most lucrative (healthiest) counties in which to offer their plans—counties without coverage are being subject to rationing. High deductibles and co-payments on insurance plans are, ultimately, a form of rationing, creating a financial stick that demands patients think carefully about the *necessity* of care.^90^ And, of course, fine-tooth comb review, acceptance, or denial of claims constitutes a case-by-case form of back-end rationing, assessing medical need on an individual—and often problematic—basis.^91,92^

This negative reality can be reimagined if aligned to a more serious combination of medicalization critique and political economy. In his pivotal discussion of medicalization, Conrad issued a challenge to sociologists: “We need to shift our attention in medicalization research and study the emergent engines of medicalization . . . . This means supplementing our social constructionist studies with political economic perspectives . . . [because] the engines that are driving medicalization have changed and we need to refocus our sociological eye as the medicalization train moves into the twenty-first century”.^10^^,pp. 11-12^ We need a more realistic assessment of the impact of economic forces encouraging medical intervention. Excessive levels of profit have a profound effect on providers’ behaviour, as the opioid crisis made all too apparent.^93,94^ Ultimately, assessing the medicalized uptake of Ozempic/Wegovy at exorbitant price levels must integrate a politico-economic understanding of how to differentiate medical need from ‘selling sickness’.^24^ Conversely, a substantive medicalization discussion, operating with a societal and not individual lens, can make a decisive contribution to our understanding of the political economy of health. Rather than considering whether individuals have made good/bad choices in relation to their health, access to medicines like Wegovy should be accompanied by a more substantive regulatory determination of the societal value of treatments in ways that look beyond current economic and profit incentives. Such a reimagining implies a more serious consideration of the broader, non-biomedical context in which medicalized conditions are situated. Targeting ‘obesity’ as disease, with one of the most overpriced drugs on the planet, decouples it from the powerful societal factors of stress, labour, inequality, food deserts, and industrialized food production. A more considerate deliberation would account for the wide array of factors that lead to biomedical interpretations in the first place, which falls in line with existing proposals to reimagine the drug development process and regulatory regime, such that both can be more innovative and responsive to public need and less oriented to the reproduction of existing, highly-profitable drug lines.^95–97^

Finally, if this substantive form of decision-making is to proceed, it suggests not only the re-evaluation of biomedical regulatory processes but also the larger health care system. Reforms limited to the drug industry will be difficult to achieve and nearly impossible to implement in a widely permissive, market-led health care system. Actors in the pharmaceutical industry make up just one pillar of a set of “industrial strategies of both purchasers and providers that defend the complexity and fragmentation of U.S. healthcare delivery, with the aim of preserving—even expanding—its profoundly accumulative nature” (^16^, p. 57). In contrast to this, unified, monopsonistic health purchasing partners price regulation with an assessment of provision in terms of societal value, and only government (state or federal) has the capacity to undertake this. In the U.S. context, the most reachable form of this is single-payer health coverage. Indeed, in outlining the aforementioned proposals for drug development and regulatory processes, Gaffney and Lexchin have underlined that, “although some of our recommendations could be implemented within the existing U.S. healthcare financing framework, full implementation would require a universal single payer system” (^96^, pk. 1039).Until Medicare Advantage plans were greatly expanded, the largest single-payer system in the world *was* U.S. Medicare. Single-payer systems, when universalized, channel costs through a single regulator, offering to providers the only price available, which they can choose to take or leave. In such systems, medicalization (and rationing) considerations play, *perforce*, an integral role in determining the boundaries of medical procedures and the estimation of their value for society.^84^ Medical providers, pharmaceuticals included, typically have no problem taking the resultant prices, as it affords them access to the health care purchasing of millions under care across the OECD world. Critics can brand it ‘rationing’ in a derogatory manner, but the comparative results speak for themselves—lower costs, broader access, consistently better health outcomes.^98^

## Conclusion

The HELP hearings exemplified how Republicans have waded into the sociological territory of medicalization, questioning the extent to which expensive drug provision should extend into American citizens’ lives. For their part, Democratic strategies to shame corporate figures on drug costs met with an equally forceful and dogmatic projection of industry's heroic morality in relation to patients’ needs. Both cases fail to grapple with the reality that capital is structurally—not morally—incentivized. Unintentionally, the Republicans’ fascination with medicalization feasibly opens the door to political economy, where a government-led (or sanctioned) evaluation of medical need could determine the value of each medical intervention. The Inflation Reduction Act, with price negotiations on a limited number of drugs, may have taken the first steps towards such an intevention, but the Trump administration seeks to slow down and undermine the implementation of this legislation. Ultimately, only health care reform, particularly a move to a single payer system, would create a purchasing and provision environment more conducive to realistic drug evaluation and cost control.^99^ Without this structural understanding of purchasing and provision, as well as a recognition of government's valuable monopsonistic role, the prioritization of medical *need* in U.S. society over profitable provision will remain a distant possibility.
